# Computing tumor trees from single cells

**DOI:** 10.1186/s13059-016-0987-z

**Published:** 2016-05-26

**Authors:** Alexander Davis, Nicholas E. Navin

**Affiliations:** Department of Genetics, The University of Texas MD Anderson Cancer Center, Houston, TX 77030 USA; Graduate School of Biomedical Sciences, The University of Texas MD Anderson Cancer Center, Houston, TX 77030 USA; Department of Bioinformatics and Computational Biology, The University of Texas MD Anderson Cancer Center, Houston, TX 77030 USA

## Abstract

Computational methods have been developed to reconstruct evolutionary lineages from tumors using single-cell genomic data. The resulting tumor trees have important applications in cancer research and clinical oncology.

Please see related Research articles: http://genomebiology.biomedcentral.com/articles/10.1186/s13059-016-0929-9 and http://genomebiology.biomedcentral.com/articles/10.1186/s13059-016-0936-x.

## Introduction

Tumors evolve from single cells. As they evolve, clonal lineages begin to diverge, resulting in distinct subpopulations and intratumor heterogeneity. This genomic diversity fuels tumor growth and enables the population of cells to survive various selective pressures in the tumor microenvironment (such as pH, hypoxia, therapy, and immune surveillance). Intratumor heterogeneity is generally considered to be ‘bad news’ from a clinical standpoint because it complicates the diagnosis and therapeutic treatment of cancer patients. However, from the perspective of an evolutionary biologist, intratumor heterogeneity provides a permanent record of the mutations that occurred during tumor growth, providing a window into time. In the same way that evolutionary biologists can infer the ancestral lineages of species that are living on our planet today, so too can cancer biologists infer the evolutionary history of a tumor by comparing clones from a single patient sample. SCITE (single cell inference of tumor evolution) and OncoNEM (oncogenetic nested effects model) [1, 2] are two statistical methods that have recently been developed for reconstructing tumor lineages from single-cell data. The trees resulting from these methods will greatly benefit studies of tumor evolution and are likely to have clinical applications in the diagnosis and therapeutic targeting of cancer patients.

## Tumor lineages

**Fig. 1:**
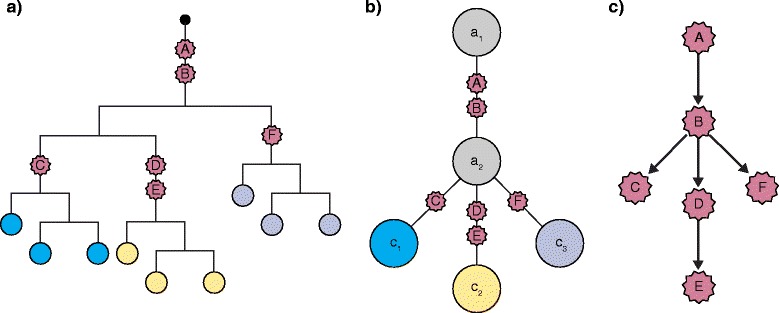
Classes of trees used in tumor phylogenetics. **a** Phylogenetic tree of single cells or macrodissected spatial samples from the tumor. **b** Clonal lineage tree with inferred common ancestors displayed in *grey*. **c** Mutational tree showing the order of mutations that occurred during tumor growth

Tumor lineages are typically represented as tree structures, of which there are three major classes: 1) phylogenetic trees, 2) clonal lineages, and 3) mutational trees (Fig. 1). Phylogenetic trees show all of the samples (or single cells) that were sequenced as leaves on the terminal nodes. Clonal lineages are condensed trees that display the lineages of the major clones and the inferred common ancestors that occurred during tumor growth. By contrast, mutational trees order the chronology of mutations that occurred during tumor growth and do not display the clones or cells associated with those mutations.

## Resolving intratumor heterogeneity

To resolve intratumor heterogeneity, several different experimental methods have been developed, including deep-sequencing, multi-region sequencing, and single-cell DNA sequencing. Deep-sequencing involves sequencing the genome or exome of a bulk tumor sample at high coverage depth and clustering mutation frequencies to identify groups of clones. This method is experimentally straightforward, but is confounded by copy-number events and overlapping frequencies of clones. Multi-region sequencing involves sampling different macroscopic regions of the tumor mass, from which the data are compared to infer a phylogenetic lineage. The limitation of this method is that it cannot distinguish clones that are intermixed within the same spatial region and is often clinically unfeasible. A limitation of both of the aforementioned methods is that they analyze bulk sequencing data that are a complex admixture of millions of different tumor cells. To deconvolute these datasets, several computational methods have been developed that use mutation frequencies to estimate the number of clonal subpopulations in the tumor mass [3]. Other statistical methods have taken one step further and use these data to infer the clonal lineages and common ancestors that occurred during tumor growth [4, 5].

## Single-cell DNA sequencing

**Fig. 2:**
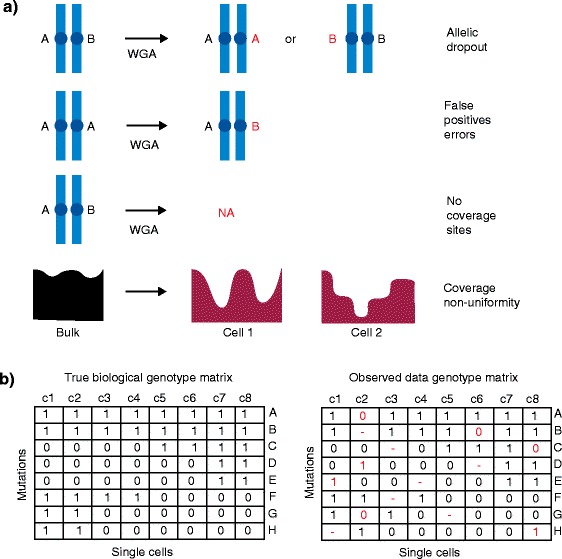
Technical errors in single-cell DNA sequencing. **a** Technical errors that occur in single-cell sequencing datasets upon whole-genome amplification, including allelic dropout, false-positive errors, genomic sites with no coverage, and coverage non-uniformity. **b** A true biological genotype matrix of single-cell mutations compared with the observed genotype matrix after technical errors are introduced during single-cell sequencing experiments. *WGA* whole-genome amplification

Another method for reconstructing phylogenetic lineages and resolving clonal subpopulations is single-cell DNA sequencing (SCS). SCS provides the highest possible resolution for resolving clonal subpopulations and can report the precise combination of mutations that occur in any given clone or cell. However, SCS datasets are limited by the total number of cells that can be sampled at a reasonable cost and extensive technical errors that arise during whole-genome amplification (WGA). This process is necessary in order to amplify a sufficient DNA quantity from a single cell to perform next-generation sequencing (NGS). The most common WGA errors include: allelic dropout (ADO) errors, false-positive errors (FPs), coverage non-uniformity, and low-coverage sites (Fig. 2). These errors introduce missing values, incorrect genotypes, and false positives into the resulting genotype matrix of single cells that is used for subsequent phylogenetic analysis. Such errors limit the direct application of phylogenetic methods that have been used in species evolution (e.g., maximum parsimony, Bayesian inference and maximum likelihood).

## OncoNEM and SCITE

SCITE and OncoNEM are computational tools that were specifically designed to address technical errors in SCS data during phylogenetic inference [1, 2]. SCITE infers a mutation tree from the single-cell sequencing data, using a Markov chain Monte Carlo (MCMC) algorithm. OncoNEM infers a clonal lineage tree from the SCS data, using a heuristic search for a phylogenetic tree, then clustering cells into subclones and inferring unobserved ancestral clones. In contrast to classical phylogenetic methods (e.g., maximum parsimony), both OncoNEM and SCITE allow a mismatch between the inferred history and the empirical data. This is accomplished using a probabilistic model of the technical errors. The methods search for the tree with the highest likelihood, defined as its probability of producing the data under this model. The model requires specifying parameters for the exact technical error rates of the single-cell sequencing process. However, both methods can estimate these parameters directly from the data and therefore do not require the user to specifically set these values. The probabilistic approach used by SCITE and OncoNEM has previously been used for phylogenetic inference of species evolution to handle sequencing error [6] and degradation of ancient DNA [7]; however, these earlier methods use different error models that are not appropriate for SCS data owing to their unique technical error profiles. In both SCITE and OncoNEM, the authors applied their methods to simulated datasets and experimental SCS datasets from human tumors, including an estrogen receptor breast cancer, invasive bladder cancer, and essential thombocymia, to evaluate their performance and benchmark their methods.

## Research and clinical applications

Tumor phylogenies have several important applications in cancer research and clinical oncology. In addition to delineating the clonal substructure of a tumor, these methods provide an inferred chronological order of mutations that occurred during tumor growth, which can be traced to understand which mutations are involved in early tumorigenic processes (e.g., initiation, angiogenesis, and invasion) compared with late cancer processes (e.g., metastasis and therapy resistance). Tumor trees can also address questions of whether tumors evolve from a single normal initiating cell, or alternatively from multiple initiating cells, as would be predicted from a mutagenic field effect. Tumor trees are also very useful for resolving clonal dynamics to improve our understanding of whether mutations occur in early punctuated bursts [8], or alternatively through the gradual accumulation of mutations over time. Phylogenetic trees can also help resolve highly controversial models that have been proposed, such as ‘neutral evolution’ which posits that tumors are not under any selection during tumor growth, leading to highly branched tree structures with no apparent clonal substructure [9]. In the clinic, phylogenetic trees of tumors will have direct applications in estimating the total amount of genomic heterogeneity in a patient’s tumor. This diversity index is expected to have prognostic value for predicting which patients will have poor response to therapy, high risk of relapse, or poor survival [10]. Tumor lineages can also be used to inform targeted therapy by directing treatment towards ‘trunkal mutations’ that occur early in the lineages and are subsequently inherited by all clones in the tumor mass. Alternatively, oncologists can use the phylogenetic trees to identify the most malignant subpopulations and target these clones independently of the other lineages.

## Notable limitations

An important limitation of both SCITE and OncoNEM is that they make the ‘infinite sites assumption’, which implies that mutations occur only once at a specific nucleotide site and are never reversed. This assumption is of course false in human tumors, where genome-wide aneuploidy is common and often results in loss of heterozygosity (LOH) and hemizygous deletions that can eliminate mutations and reverse the genotype. Another violation of this assumption is convergent evolution, in which activating driver mutations can occur at the same nucleotide site in independent clones or lineages owing to strong positive selection. An additional criticism of both studies is that there is no straightforward way to know whether the clonal lineages or mutational trees generated from the human tumors truly reflect the biological lineages in any way. This is admittedly a very difficult problem to address and would require a ‘gold-standard’ tumor lineage to which different methods can be benchmarked to determine their accuracy. These experiments would be difficult to perform on a human tumor sample (owing to limited tissue), but might be feasible in artificial lineages that are generated in cell culture systems that are passaged for many generations, which are intentionally diverged and documented.

## Concluding remarks

SCITE and OncoNEM are innovative statistical methods that address a crucial problem in the construction of phylogenetic lineages from SCS data by using error models. These methods were shown to improve the accuracy of clonal lineages and mutational trees, as demonstrated in simulated datasets and human tumor samples. New technologies are on the horizon that will soon increase the throughput of DNA SCS to thousands of single cells, which will improve the accuracy of tumor lineages. OncoNEM and SCITE are well prepared to analyze these large-scale datasets and greatly facilitate the interpretation of the resulting lineages. Future versions of OncoNEM and SCITE could be improved in several ways. Removing the infinite sites assumption will be an important next step, which has already been addressed in classical phylogenetic methods such as maximum parsimony. Another important step will be to incorporate single-cell copy number and LOH into the tumor lineages, which can lead to missing mutations in the genotype matrix and violate the perfect phylogenies. Another avenue of progress will be to improve the probabilistic error models. Both methods currently assume a fixed probability of error at every site, but could instead assume higher error probabilities for lower-confidence mutation calls. However, even without these improvements, SCITE and OncoNEM are a major step forward in the analysis of SCS data and will have immediate applications in both cancer research and medicine.

## Abbreviations

LOH, loss of heterozygosity; MCMC, Markov chain Monte Carlo; OncoNEM, oncogenetic nested effects model; SCITE, single cell inference of tumor evolution; SCS, single-cell DNA sequencing; WGA, whole-genome amplification.
